# Highly Effective Non-Viral Antitumor Gene Therapy System Comprised of Biocompatible Small Plasmid Complex Particles Consisting of pDNA, Anionic Polysaccharide, and Fully Deprotected Linear Polyethylenimine

**DOI:** 10.3390/pharmaceutics7030152

**Published:** 2015-07-23

**Authors:** Yoshiyuki Koyama, Kikuya Sugiura, Chieko Yoshihara, Toshio Inaba, Tomoko Ito

**Affiliations:** 1 Japan Anti-tuberculosis Association, Shin-Yamanote Hospital, 3-6-1 Suwa-cho, Higashimurayama, Tokyo 189-0021, Japan; E-Mail: tomoko_ito@nifty.com; 2 Graduate School of Life and Environmental Sciences, Osaka Prefecture University 1-58 Rinku-oraikita, Izumisano, Osaka 598-8531, Japan; E-Mails: sugiura@vet.osakafu-u.ac.jp (K.S.); inaba@vet.osakafu-u.ac.jp (T.I.); 3 Department of Home Economics, Otsuma Women’s University, 12 Sanbancho, Chiyoda-ku, Tokyo 102-8357, Japan; E-Mail: yoshihara@otsuma.ac.jp

**Keywords:** non-viral, transfection, plasmid, Polyethylenimine “Max”, freeze-drying, hyaluronic acid, chondroitin sulfate, antitumor, cytokine, nanoparticle

## Abstract

We have reported that ternary complexes of plasmid DNA with conventional linear polyethylenimine (l-PEI) and certain polyanions were very stably dispersed, and, with no cryoprotectant, they could be freeze-dried and re-hydrated without the loss of transfection ability. These properties enabled the preparation of a concentrated suspension of very small pDNA complex, by preparing the complexes at highly diluted conditions, followed by condensation via lyophilization-and-rehydration procedure. Recently, a high potency linear polyethylenimine having no residual protective groups, *i.e.*, Polyethylenimine “Max” (PEI “Max”), is available, which has been reported to induce much higher gene expression than conventional l-PEI. We tried to prepare the small DNA/PEI “Max”/polyanion complexes by a similar freeze-drying method. Small complex particles could be obtained without apparent aggregation, but transfection activity of the rehydrated complexes was severely reduced. Complex-preparation conditions were investigated in details to achieve the freeze-dried DNA/PEI “Max”/polyanion small ternary complexes with high transfection efficiency. DNA/PEI “Max”/polyanion complexes containing cytokine-coding plasmids were then prepared, and their anti-tumor therapeutic efficacy was examined in tumor-bearing mice.

## 1. Introduction

Considerable efforts have been invested in developing non-viral vector systems as safer alternatives to live viruses [1]. However, the *in vivo* gene expression by such artificial vectors is strictly limited due to the low delivery efficiency to the target cells [2]. One of the major obstacles to efficient *in vivo* transfection is the non-specific adverse interactions of the plasmid DNA (pDNA) complexes with unintended bio-components [3]. Since the surfaces of pDNA/polycation complexes usually have a cationic charge, they strongly interact with blood cells, proteins, or extracellular matrices. Another significant barrier to *in vivo* gene delivery is too large size of the pDNA complexes. If a single pDNA molecule forms one complex particle, it should have a diameter of less than 40 nm [4]. However, such small particles can be generated only at highly diluted conditions. Common conditions for preparation of the plasmid complex for *in vivo* transfection (>200 μg/mL) always afford particles with much larger diameter (>500 nm) [5]. Moreover, they readily aggregate to form further large particles, and often precipitate [6].

In order to achieve efficient *in vivo* delivery, we employed the following strategies: First we searched for anionic polymers that could be deposited onto pDNA/polycation complexes without decomposition of the preformed complexes, and could recharge them to make them anionic. Then we identified certain polyanions, such as PEG derivatives having carboxyl side chains [7,8,9], and hyaluronic acid (HA) [5,10,11,12] that could protectively coat the pre-formed pDNA/polycation complexes and generate stable anionic particles. These complexes did not interact with serum proteins [10] or red blood cells [5].

Polyanion-coated pDNA/polycation complexes (DNA/polycation/polyanion ternary complexes) showed high dispersibility, and they stably dispersed not only in pure water, but in high-salt solutions, such as saline, phosphate buffered saline (PBS), or cell culture medium. They did not aggregate, but were stably suspended for hours in water or other media [13]. This high stability of the pDNA/polycation/polyanion ternary complex makes it possible to attain very small complex particle preparations.

We mixed pDNA with hyaluronic acid, and then linear polyethylenimine (l-PEI), in the given order, as pre-addition of the polyanion had been found to afford complexes of smaller size than those obtained by post-addition of the polyanion to the pre-formed pDNA/polycation complexes [5]. Preparation of these complexes was performed under very dilute conditions ([pDNA] = 6 μg/mL). At such a low concentration, most complexes formed were under 70 nm in diameter. They were then freeze-dried, and re-hydrated to concentration of 200 μg/mL. Finely suspended complexes with size and size-distribution comparable with the original suspension were obtained. They showed high transfection efficiency comparable with that of the freshly prepared complexes before freezing. The re-hydrated very small complexes (~70 nm) at a suitable concentration for *in vivo* transfection (200 μg/mL) showed fairly high gene expression in tumor after intravenous or intra-tumor injections [11].

These experiments were carried out using conventional l-PEI, which contains *N*-propanoyl group in up to 7%–8% of the repeating units. Recently, a high potency linear PEI having no residual protective groups, Polyethylenimine “Max” (PEI “Max”), has become available, which was reported to mediate much higher gene expression in the cells than conventional l-PEI with protective group residues. We tried to prepare the pDNA/(PEI “Max”)/polyanion complexes by a similar procedure. Mixing of pDNA, HA, and PEI “Max” in this order at [pDNA] = 6 μg/mL generated fairly small stable complex particles. They showed significantly higher (70-fold) gene expression than achieved by conventional l-PEI in cultured cells, as was expected. However, lyophilization of the pDNA/(PEI “Max”)/HA complex suspension produced a highly shrunken white sponge, which could not be re-suspended in water. It remained undissolved for more than five days.

In this study, we explored the optimum conditions for preparation of the freeze-dried pDNA/(PEI “Max”)/polyanion ternary complexes, which could be re-hydrated easily into fine suspensions having high activity for *in vivo* applications. Highly effective pDNA/(PEI “Max”)/polyanion complexes containing cytokine-coding plasmid were prepared, and their anti-tumor therapeutic efficacy was examined in tumor-bearing mice.

## 2. Experimental Section

### 2.1. Materials and Mice

Hyaluronic acid sodium salt from chicken combs (MW 280,000) (HA) and chondroitin sulfate sodium salt from shark cartilage (MW 10,000) (CS) and were supplied by Seikagaku Corp. (Tokyo, Japan). Dextran (MW 180,000–220,000) was given by MRC Polysaccharides Co., Ltd. Polyethylenimine, Linear (l-PEI; MW 25,000 in a free base form), and Polyethylenimine “Max” (MW 40,000), High Potency Linear PEI, (PEI “Max”; MW 40,000 in a hydrochloride salt form, comparable to linear polyethylenimine of MW 25,000 which is not in the hydrochloride salt form) was purchased from Polyscience, Inc. (Warrington, PA, USA). Plasmid DNA (7.0 kbp) containing firefly luciferase gene under the control of a cytomegalovirus promoter was a kind gift from Prof. Kawakami (Nagasaki University, Nagasaki, Japan). Murine granulocyte macrophage colony-stimulating factor (GM-CSF) encoding pDNA with cytomegalovirus promoter was a kind gift from Prof. Hamada (Ehime University, Ehime, Japan). Amplification of the pDNAs was performed by AMBiS Corporation (Okinawa, Japan). Cell culture lysis reagent and luciferase assay substrate were purchased from Promega Corporation (Madison, WI, USA). The protein assay kit was obtained from Bio-Rad Laboratories (Hercules, CA, USA). Male C57BL/6- and ddY-mice (5 weeks old) were purchased from Tokyo Laboratory Animals Science Co., Ltd. All the animal studies were carried out in accordance with the guidelines of Otsuma Women’s University (certificate numbers: 10004, 121004 and 13005).

### 2.2. ζ-Potential and Size Measurement

Plasmid DNA complexes were prepared under the same conditions with those for *in vitro* or *in vivo* transfection. They were diluted with water, and applied to a particle analyzer (MALVERN Zetasizer Nano ZS, Malvern, Worcestershire, UK).

### 2.3. In Vitro Transfection

#### 2.3.1. Preparation of pDNA Complex for *in Vitro* Transfection

Typically, an aqueous pDNA solution (1.25 μg in 31.3 μL; [P] = 121 μM) was mixed with 62.5 μL of HA solution ([COOH] = 728 μM), or CS solution ([COOH] + [S] = 485 μM). It was then mixed with 31.3 μL of a l-PEI or PEI “Max” solution ([N] = 1.45 mM). The solution was allowed to stand for 20 min, after which a dextran solution (1.25 μL; 10%) was added. The mixture was then frozen at −20 °C, and freeze-dried at room temperature. The resulting white spongy DNA complex was rehydrated with 125 μL of water or a buffered solution just before use, and pH and salt strength were adjusted to physiological conditions prior to addition to the cells. The charge ratio of this preparation was DNA:PEI:HA = 1:12:12, and DNA:PEI: CS = 1:12:8.

#### 2.3.2. *In Vitro* Transfection

Plasmid DNA encoding luciferase was used to measure the gene expression efficiency. B16 cells, a mouse melanoma cell line, were seeded in 24-well plates at 2.6 × 10^4^ cells per well, and cultured in 1 mL minimum essential medium (MEM) supplemented with 10% fetal bovine serum (FBS), penicillin G sodium (100 unit/mL), and streptomycin sulfate (0.1 mg/mL) for 2 days. The medium was then replaced with 1 mL of fresh medium with FBS and the antibiotics. DNA complex suspension containing 1.25 μg of pDNA prepared above was added to each well, and the cells were incubated at 37 °C in a 5% CO_2_ incubator. After 1 day, the cells were harvested, lysed, and assessed by a Pica Gene luciferase assay kit (TOYO INK Co., Ltd., Tokyo, Japan). Further, protein levels of the lysate were estimated by protein assay kit (Bio-Rad Laboratories, Inc., Hercules, CA, USA).

### 2.4. In Vivo Imaging

#### 2.4.1. Preparation of Plasmid Complex for *in Vivo* Imaging

CS (1190 μg) in phosphate buffer (pH 7.4; 7.4 mM, 4740 μL) and PEI “Max” (587 μg in 117 μL) were added in this order to an aqueous solution of pDNA encoding luciferase (200 μg in 146 μL). The solution was allowed to stand for 20 min, after which the dextran solution (50 μL; 10%) was added. The mixture was then frozen at −20 °C and freeze-dried at room temperature to give a spongy DNA/(PEI “Max”)/CS complex (1:12:8 in charge). It was rehydrated with 250 μL of water just before use.

#### 2.4.2. Image Acquisition

Male C57BL/6 mice (5 weeks) were inoculated subcutaneously with 2 × 10^6^ B16 cells. When the size of the tumor reached over 3 mm in diameter, the lyophilized-and-rehydrated DNA complex suspension was injected intratumorally (DNA: 200 μg/mouse). Ten minutes before recording observations, the mice were anesthetized, and injected intraperitoneally with d-luciferin (7.5 mg in 150 μL PBS). The images of whole body were obtained using a Night-OWL LB 981 NC 320 instrument (Berthold Technologies GmbH, Bad Wildbad, Germany). The pseudocolor luminescent images were generated and overlaid on the whole body images using WinLight software (Berthold Technologies GmbH).

### 2.5. Treatment of Cancer

#### 2.5.1. Preparation of Plasmid Complex for *in Vivo* Transfection

To examine the antitumor efficacy of the pDNA complex, pDNA/(PEI “Max”)/CS (or HA) ternary complexes (1:12:12 in charge) were prepared similarly as for *in vivo* imaging from the plasmid encoding GM-CSF. The complex containing 100 μg of the plasmid re-suspended in 250 μL phosphate buffer (pH 7.4) was prepared briefly as follows: Plasmid DNA (100 μg), CS (889 μg) (or HA, 1460 μg), and PEI “Max” (294 μg) were mixed in phosphate buffer (pH 7.4; 7 mM, 5 mL). Dextran (125 μL; 4%) was added, and the mixture was frozen-and-lyophilized. It was rehydrated with 250 μL of water just before administration.

#### 2.5.2. Evaluation of the Therapeutic Effect of the pDNA Complexes

B16 cells were injected subcutaneously into ddY mice (male, 5 weeks) (4.1 × 10^5^ cells per mouse). After one week, lyophilized-and-rehydrated pDNA/(PEI “Max”)/HA (or CS) ternary complex containing 100 μg of the plasmid was injected intratumorally five times every other day, and the tumor size was monitored for 6 weeks (*n* = 3).

## 3. Results and Discussion

### 3.1. Effect of Mixing Ratio of DNA and PEI “Max”

**Figure 1:**
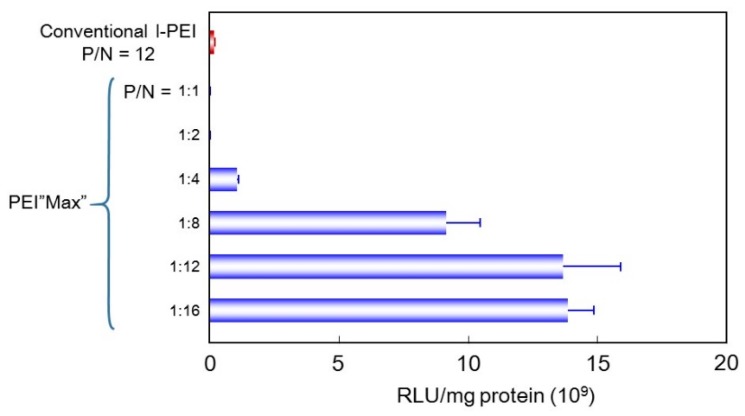
Effect of mixing ratio of DNA and PEI “Max” on the transfection efficiency.

Plasmid coding luciferase was mixed with PEI “Max” at various ratios to select the most suitable ratio for gene transfection. Freshly prepared complexes were added to cultured B16 cells, and after two days, luciferase activity was measured (Figure 1). At DNA:(PEI “Max”) charge ratio (N/P ratio) = 8–16, they showed significantly higher (about 80-fold) gene expression than that with conventional l-PEI in the cultured cells, as expected. The complex prepared at N/P = 12 showed higher gene expression than those prepared at lower N/P ratio. At N/P = 16, even higher expression level was sometimes obtained. However, at this relatively high N/P ratio, slight cytotoxicity was observed. The DNA:(PEI “Max”) ratio was then fixed to 1:12 in charge.

### 3.2. Effect of Dextran Addition on Rehydration Property

**Figure 2:**
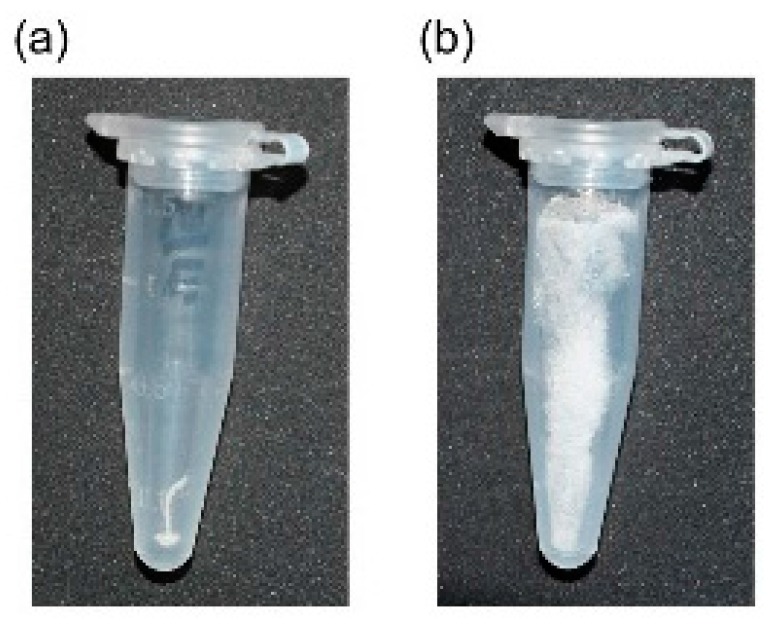
DNA/(PEI “Max”)/HA ternary complexes freeze-dried under (**a**) absence or (**b**) presence of 0.1% dextran.

**Figure 3:**
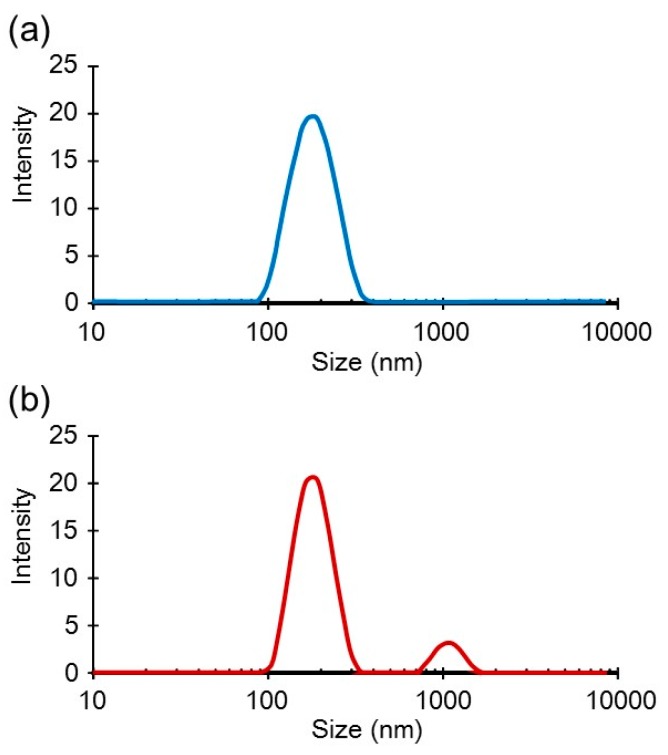
Size distribution profiles of the DNA/(PEI “Max”)/HA complexes (1:12:12) (**a**) before and (**b**) after lyophilization under the presence of 0.1% dextran.

As per earlier report [14], addition of HA, and l-PEI to pDNA in this order at final [DNA] = 6 μg/mL (mixing ratio was DNA:PEI:HA = 1:12:12 in charge) gave stable suspension of the small complex particles. They were lyophilized to be a soft spongy material, which could be rehydrated to reproduce fine complex particles without loss of transfection activity. PEI “Max” also afforded small stable complex particles by mixing with pDNA and HA in the similar conditions (Figure 3a). However, Freeze-drying of the pDNA/(PEI “Max”)/HA complex suspension resulted in a highly shrunken white sponge (Figure 2a), which could not be rehydrated nor re-suspended in water.

**Figure 4:**
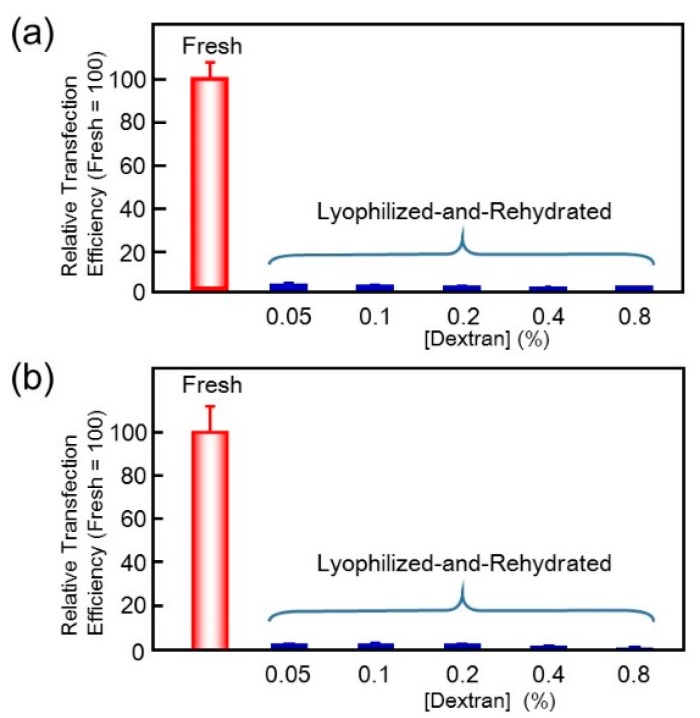
Effect of dextran concentration on gene expression levels after lyophilizaion-and-rehydration. (**a**) DNA/(PEI “Max”)/HA complexes (1:12:12); (**b**) DNA/(PEI “Max”)/CS complexes (1:12:8).

Dextran was then added to the pDNA/(PEI “Max”)/HA complex suspension before freeze-drying to facilitate rehydration. With more than 0.1% dextran, white spongy material was obtained after freeze-drying (Figure 2b). It was easily rehydrated to a suspension of the negatively charged fine particles with ζ-potential of −30 to −35 mV. A small part of the particles was aggregated to around 1 μm in diameter, while 90% of them were still finely suspended (Figure 3b), as before freeze-drying. However, transfection efficiency was not improved even with high concentration of dextran (Figure 4a). Another anionic polysaccharide, CS was then attempted in place of HA. Small complex particles were obtained similarly with CS, but freeze-drying of the pDNA/(PEI “Max”)/CS complex also afforded a shrunken sponge, which never re-dissolved again. Addition of dextran also improved the hydration behavior of the lyophilized pDNA/(PEI “Max”)/CS complex, and small particles having ζ-potential of around −35 mV were obtained. However, their transfection activity decreased to about 0.05%–2.5% regardless of the dextran concentration (Figure 4b).

### 3.3. Effect of Mixing Ratio of HA (or CS)

**Figure 5:**
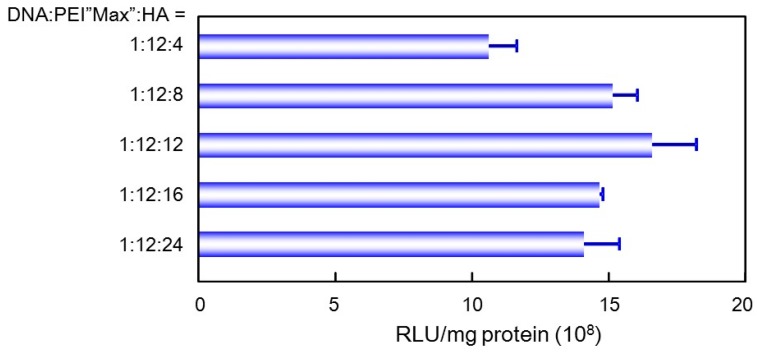
Effect of mixing ratio of hyaluronic acid on the gene expression activity.

Plasmid encoding luciferase was mixed with PEI “Max” and HA (or CS) at various ratios to ascertain a suitable mixing ratio for gene transfection. After freeze-drying in the presence of 0.1% dextran, the gene expression activity was evaluated. The amount of HA in the pDNA/(PEI “Max”)/HA ternary complex much affected the transfection efficiency, and the rehydrated complex at DNA:(PEI “Max”):HA = 1:12:12 expressed highest luciferase activity (Figure 5). As for the complexes with CS, gene expression activity was also influenced by the mixing ratio of CS, and those prepared at DNA:(PEI “Max”):CS = 1:12: 8, and 1:12:12 showed almost the same highest level of gene expression (data not shown).

### 3.4. Effect of Salt Strength and pH of the Rehydrating Solution

**Figure 6:**
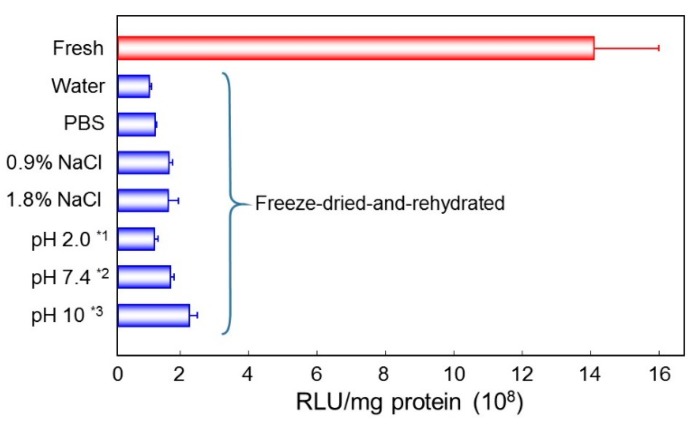
Gene expression by the freeze-dried DNA/(PEI “Max”)/CS complexes (1:12:8) rehydrated with various solutions. *^1^ Glycine hydrochloric acid buffer; *^2^ Phosphoric acid buffer (PB); *^3^ Glycine sodium hydroxide buffer.

Freeze-dried complex was rehydrated by solutions of various salt strength or pH values. Lyophilized DNA/(PEI “Max”)/CS complex (1:12:8) was rehydrated with water, phosphate buffered saline (PBS), 0.9% NaCl, 1.8% NaCl, or 10 mM buffers of pH 2.0, 7.4, or 10. Before adding to cultured cells, they were adjusted to physiological osmolality and pH. High salt strength rather decreased the transfection efficiency. On the other hand, rehydration under neutral or weak alkaline conditions gave the suspension with relatively high activities (Figure 6). Rehydration at pH 10 induced highest transgene expression among them, but it is still 15% of that of the freshly prepared original complex.

### 3.5. Effect of Salt Strength and pH of the Complex Preparation Conditions

**Figure 7:**
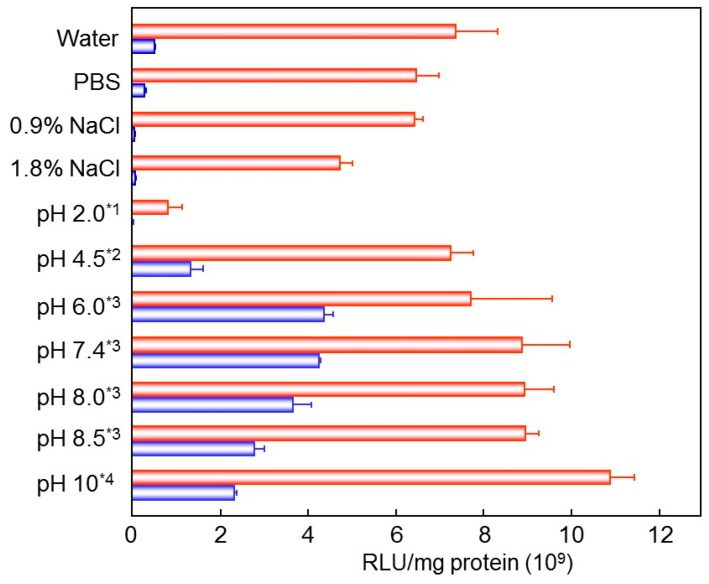
Gene expression profiles of the freeze-dried DNA/(PEI “Max”)/CS complexes (1:12:8) prepared in various solutions. *^1^ Glycine hydrochloric acid buffer; *^2^ Acetic acid buffer; *^3^ Phosphoric acid buffer (PB); *^4^ Glycine sodium hydroxide buffer. Red bars stand for the results with freshly prepared complex, and blue bars stand for those with lyophilized-and-rehydrated complexes.

Gene transfection activity was slightly improved by the pH value in the rehydration process, but was not satisfactory enough to attain high transfection efficiency. Effect of salt strength and pH value at the complex formation was then evaluated. DNA/(PEI “Max”)/CS complex (1:12:8) was prepared in water, PBS, 0.9% NaCl, 1.8% NaCl, or 10 mM buffers of various pH values. They were lyophilized, and then rehydrated with pure water. Osmotic pressure was adjusted to physiological conditions before administration. DNA/(PEI “Max”)/CS complex prepared in 0.9% NaCl, or 1.8% NaCl showed rather lower gene expression than that prepared in pure water or in PBS. On the other hand, pH value of the preparation solution gave a strong influence. The complex prepared at pH 2.0 showed almost no transfection activity after rehydration, while those made at pH 6–7.4 demonstrated improved efficiency, approximately eight times higher gene expression than that obtained in water (Figure 7). The most appropriate condition, so far, for preparation of lyophilized DNA:(PEI “Max”):CS complex was mixing the components in phosphate buffer at pH 6–7.4, followed by freeze-drying in presence of 0.1% dextran. DNA complexes with HA were also prepared under similar conditions and examined for their transfection efficiency after rehydration. White spongy freeze-dried DNA:(PEI “Max”):HA complex could be easily re-hydrated into finely suspended particles, which had almost the same gene expression efficiency (93%) as the DNA:(PEI “Max”):CS complex obtained by treatment of same conditions.

In order to prepare a suspension of small pDNA complexes (~200 nm), DNA must be mixed with PEI “Max” at very low concentration ([DNA] < 50 μg/mL). If it contains enough DNA for effective therapy, the volume would be too much to be injected into a living body. It should thus be concentrated to a small volume to be administered. Therefore, the lyophilizing-rehydration procedure is necessary to prepare the small DNA complex suspension for *in vivo* transfection, although the transfection efficiency is still not comparable to that of freshly prepared complexes. Besides, rehydrated suspension should have physiological pH and ionic strength to be administered into the body. Then preparation condition of the plasmid complexes for *in vivo* use was set as follows: HA (or CS), and PEI “Max” are mixed with plasmid DNA in this order in 7 mM phosphate buffer of pH 7.4 at the final concentration of plasmid is 20–40 μg/mL. The mixing charge ratio is DNA:PEI:HA = 1:12:12, or DNA:PEI:CS = 1:12:8 (or 12). Allowing the solution to stand for 20 min, a small volume of dextran solution is added (final dextran is 0.1%), and the mixture is frozen, and freeze-dried. It is rehydrated with one-twentieth volume of pure water just before use.

### 3.6. In Vivo Imaging

**Figure 8:**
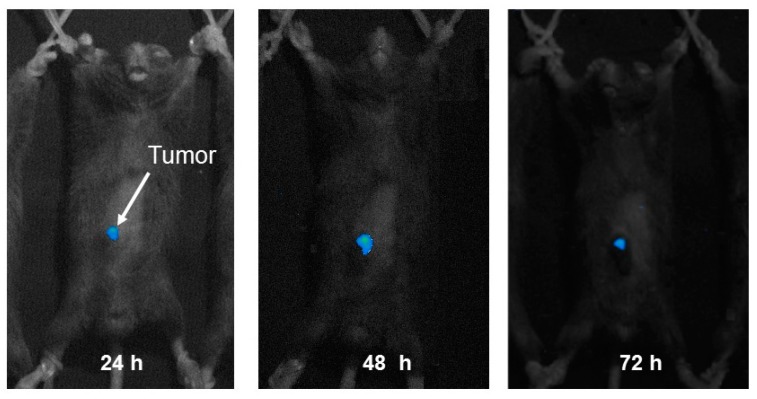
*In vivo* imaging of the tumor-bearing mouse injected with 200 μg of DNA(luciferase)/(PEI “Max”)/CS complexes (1:12:8).

The small pDNA complex suspension, prepared with the methods described above, was expected to mediate high gene transfection *in vivo*, especially in the tumor tissue. The pDNA/(PEI “Max”)/CS ternary complex was then prepared with plasmid coding luciferase, and injected into mice (200 μg of plasmid per mouse) of which B16 cells had been subcutaneously inoculated in the abdomen. The *in vivo* imaging was performed one to three days after the administration. As can be seen in Figure 8, tumor tissue clearly emits luminescent light, and the luminescence lasted over three days.

Injection volume of the complex suspension was fairly large (250 μL). It could have overflowed outside the tumor, although it was injected into the tumor tissue. Nevertheless, only tumor tissue was luminous during imaging. The plasmid complex that spread into the subcutis seemed to flow into the microcirculation of blood or lymph, and then accumulate in the tumor by the EPR effect [14]. Gene expression in tumor was also confirmed by GFP-coding plasmid DNA complex. Two days after intratumor or intravenous injection of the DNA(GFP)/(PEI “Max”)/CS complex containing 10 μg of the plasmid, up to 38% or 25% of the tumor cells were positive for GFP expression, respectively. This high expression level persistent for days in the tumor tissue, is expected to bring about the high therapeutic effect on the tumor bearing mice.

### 3.7. Antitumor Efficacy of the Small pDNA/(PEI “Max”)/HA (or CS) Ternary Complex in the Tumor Model Mice

**Figure 9:**
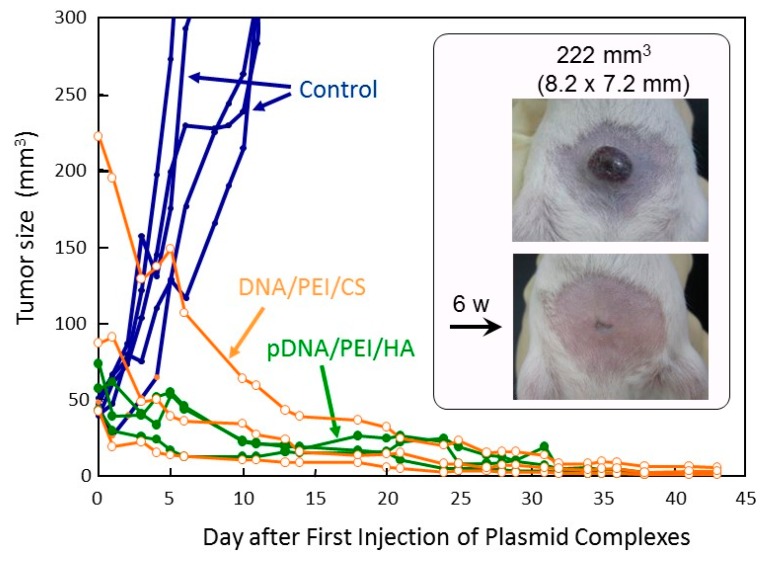
Therapeutic efficacy of DNA(GM-CSF)/(PEI “Max”)/HA (or CS) complexes (1:12:12) in tumor-bearing mice.

This tumor specific high gene expression seems to be favorable for anti-tumor gene therapy. The complex comprising plasmids coding GM-CSF was then prepared with HA or CS at the charge ratio of DNA:(PEI “Max”):HA (or CS) = 1:12:12, and injected intratumorally into tumor bearing mice. Change in tumor size was illustrated in Figure 9. Both the plasmid complexes with HA or CS showed a strong antitumor effect. In all six cases, tumor completely disappeared in 40 days, and regrowth was seen in no mouse until 100 days after the plasmid complex administration. It is known that intra-tumor injection of nucleic acids may induce innate immune reactions including cytokine production that can have profound effects on tumor growth. However, in our preliminary experiments, luciferase-transfection induced little or no tumor growth suppression. This evident tumor-suppressing efficacy observed here can be attributed to the high expression of the cytokine-gene in the tumor cells.

## 4. Conclusions

pDNA complexes prepared in phosphate buffer (pH 7.4) could be lyophilized-and-rehydrated to afford small plasmid complexes with high transfection efficiency. They showed tumor specific gene expression after injection into mice. Complexes comprising pDNA coding GM-CSF showed very high anti-tumor effect in tumor-bearing mice.

## References

[1] Glover D.J., Lipps H.J., Jans D.A. (2005). Towards safe, non-viral therapeutic gene expression in humans. Nat. Rev. Genet..

[2] Morille M., Passirani C., Vonarbourg A., Clavreul A., Benoit J.P. (2008). Progress in developing cationic vectors for non-viral systemic gene therapy against cancer. Biomaterials.

[3] Finsinger D., Remy J.S., Erbacher P., Koch C., Plank C. (2000). Protective copolymers for nonviral gene vectors: Synthesis, vector characterization and application in gene delivery. Gene Ther..

[4] Dauty E., Behr J.P., Remy J.S. (2002). Development of plasmid and oligonucleotide nanometric particles. Gene Ther..

[5] Ito T., Iida-Tanaka N., Koyama Y. (2008). Efficient *in vivo* gene transfection by stable DNA/PEI complexes coated by hyaluronic acid. J. Drug Target..

[6] Trubetskoy V.S., Loomis A., Slattum P.M., Hagstrom J.E., Budker V.G., Wolff J.A. (1999). Caged DNA does not aggregate in high ionic strength solutions. Bioconjug. Chem..

[7] Koyama Y., Yamada E., Ito T., Mizutani Y., Yamaoka T. (2002). Sugar-Containing Polyanions as a Self-Assembled Coating of Plasmid/Polycation Complexes for Receptor-Mediated Gene Delivery. Macromol. Biosci..

[8] Koyama Y., Ito T., Matsumoto H., Tanioka A., Okuda T., Yamaura N., Aoyagi H., Niidome T. (2003). Novel poly(ethylene glycol) derivatives with carboxylic acid pendant groups: Synthesis and their protection and enhancing effect on non-viral gene transfection systems. J. Biomater. Sci. Polym. Ed..

[9] Koyama Y., Yamashita M., Iida-Tanaka N., Ito T. (2006). Enhancement of transcriptional activity of DNA complexes by amphoteric PEG derivative. Biomacromolecules.

[10] Ito T., Iida-Tanaka N., Niidome T., Kawano T., Kubo K., Yoshikawa K., Sato T., Yang Z., Koyama Y. (2006). Hyaluronic acid and its derivative as a multi-functional gene expression enhancer: Protection from non-specific interactions, adhesion to targeted cells, and transcriptional activation. J. Control. Release.

[11] Ito T., Yoshihara C., Hamada K., Koyama Y. (2010). DNA/polyethylenimine/hyaluronic acid small complex particles and tumor suppression in mice. Biomaterials.

[12] Ito T., Koyama Y., Otsuka M. (2010). Analysis of the surface structure of DNA/polycation/hyaluronic acid ternary complex by Raman microscopy. J. Pharm. Biomed. Anal..

[13] Maruyama K., Iwasaki F., Takizawa T., Yanagie H., Niidome T., Yamada E., Ito T., Koyama Y. (2004). Novel receptor-mediated gene delivery system comprising plasmid/protamine/sugar-containing polyanion ternary complex. Biomaterials.

[14] Maeda H., Wu J., Sawa T., Matsumura Y., Hori K. (2000). Tumor vascular permeability and the EPR effect in macromolecular therapeutics: A review. J. Control. Release.

